# Newborn with a solitary hairless skin defect on the scalp vertex

**DOI:** 10.1002/ccr3.2468

**Published:** 2019-10-07

**Authors:** Dimitra Koumaki, Vasiliki Koumaki, Sotirios Boumpoucheropoulos, Ludmila Baltaga, Panagiotis Bitados, Alexander Katoulis, Konstantinos Krasagakis

**Affiliations:** ^1^ Paediatric Dermatology Department Chelsea and Westminster Foundation TRUST London UK; ^2^ Department of Microbiology Medical School University of Athens Athens Greece; ^3^ Department of Medical Oncology Agioi Anargyroi, General Oncological Hospital Athens Greece; ^4^ Medical School of Crete Heraklion Greece; ^5^ 2nd Department of Dermatology and Venereology National and Kapodistrian University of Athens, Medical School, “Attikon” General University Hospital Athens Greece; ^6^ Dermatology Department University Hospital of Heraklion Heraklion Greece

**Keywords:** aplasia cutis congenita, congenital disorders, newborn, scalp

## Abstract

Aplasia cutis congenita is a rare congenital disorder usually presenting as an isolated lesion on the scalp that may be associated with genetic syndromes and congenital anomalies. Therefore, it is important to be aware of this syndrome.

## Graphical abstract

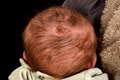

## 1 CLINICAL IMAGE

**Figure 1:**
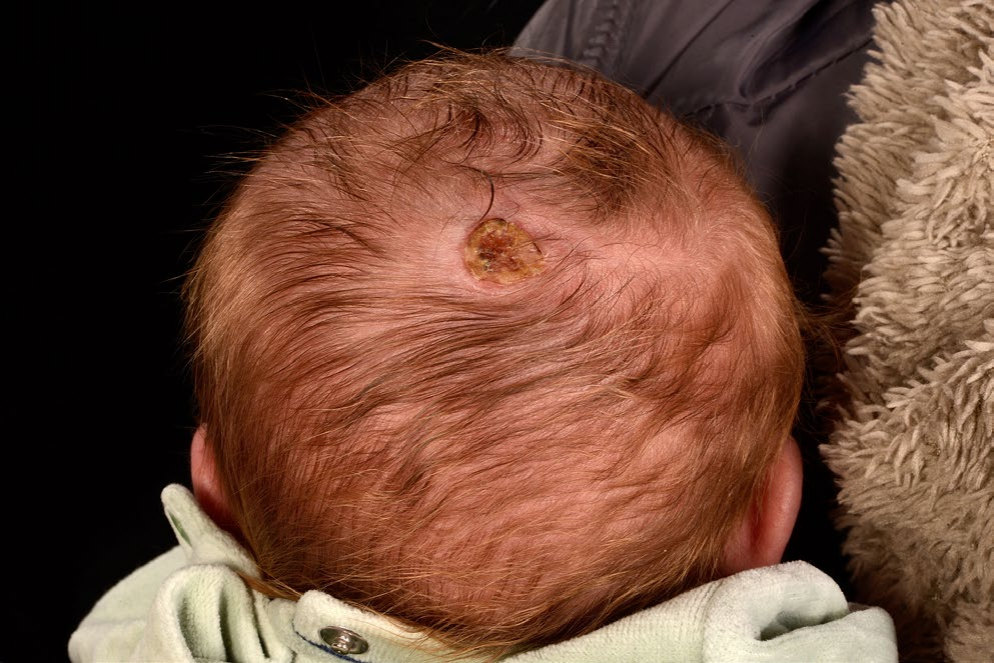
Solitary, hairless crusted skin defect on the scalp vertex

**Figure 2:**
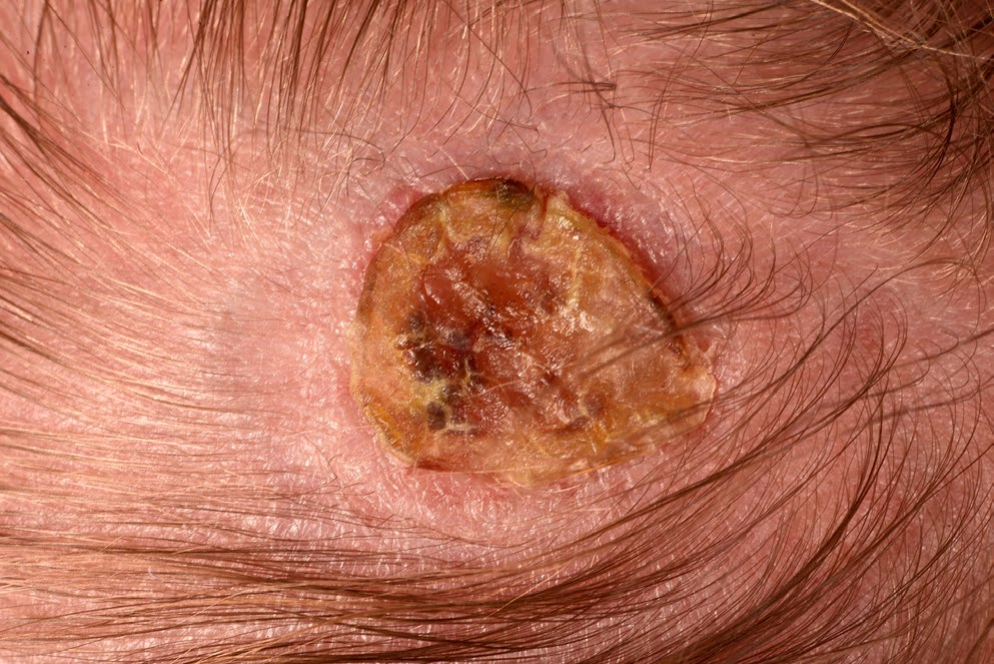
Orangish oval lesion with punched‐out appearance on the posterior part of the scalp

**Figure 3:**
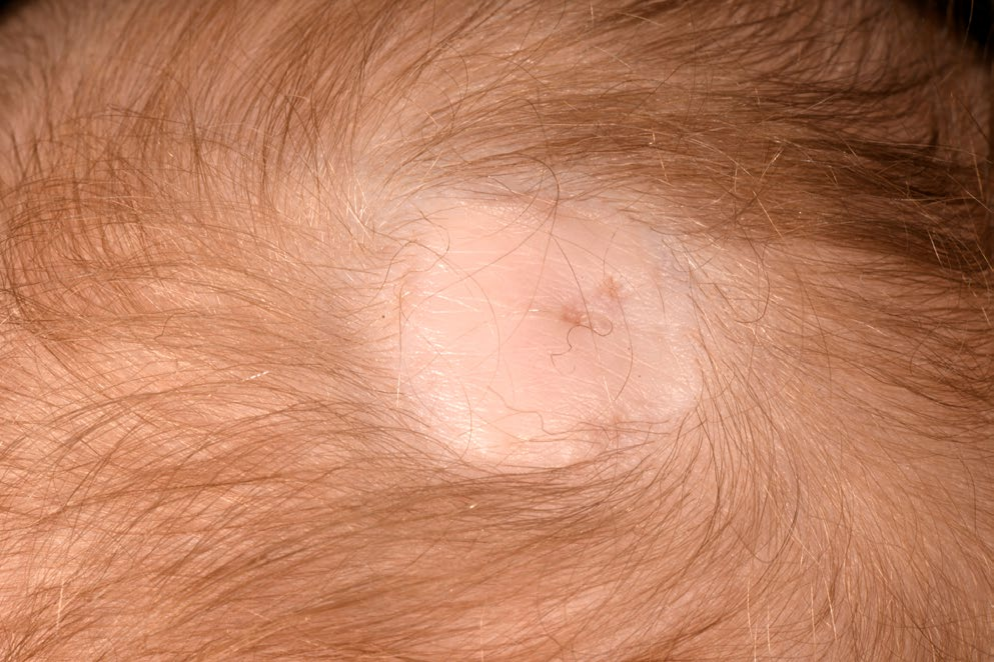
Oval whitish scar‐like plaque after 2 mo of follow‐up

A 2‐day‐old term male newborn was referred for dermatology review for a solitary lesion on the posterior part of his scalp that was presented at birth. He had an uncomplicated pregnancy. On clinical examination, there was a hairless well‐demarcated oval ulcerated crusted orangish plaque with rolled edges on the vertex of the scalp (Figures 1 and 2). After 2 months on his follow‐up appointment, the lesion on the scalp has healed up completely with scarring (Figure 3). The child was neurodevelopmentally normal. What is the diagnosis?

## 2 ANSWER

The diagnosis was aplasia cutis congenita (ACC). ACC is a rare, heterogeneous group of congenital disorders of unknown etiology that is characterized by focal or widespread absence of the skin present at birth or shortly after.^1^ Its precise incidence is unknown but has been thought to be around 1 to 3 in 10 000 live births.^1^ Approximately 85 percent of ACC cases manifest as isolated lesions on the scalp.^2^ The diagnosis of ACC is clinical, and the appearance is highly variable. The physical examination shows ulcerations or erosions of the skin that may extend to deeper tissues, such as the muscle or bone. Clinicians should obtain a complete family and birth history with particular attention to any perinatal trauma, illnesses, medications, or family members with either ACC or limb defects. Although in most of the cases, as in our case, ACC is an isolated finding with good prognosis healing up with scarring within few months,^2^ it also may be a feature of a variety of genetic syndromes, such as trisomy 13, 4p deletion syndrome, epidermolysis bullosa (EB), ectodermal dysplasias, Adams‐Oliver syndrome (AOS), and amniotic band sequence.^1^ Therefore, in patients who have any additional dysmorphic features, array cytogenetic testing should be carried out as some cases are associated with genetic abnormalities.^1^, ^2^

## CONFLICT OF INTEREST

None declared.

## AUTHOR CONTRIBUTIONS

DK was involved in conception or design of the work, reviewing the patient, arranging for the clinical images, and writing up the article. VK, SB AK, LB, PB, and KK were involved in critical revision of the article. All authors contributed to the final version of the manuscript.

## CONSENT STATEMENT

Informed written consent was obtained from the patient's guardian for publication of the images.
